# Electroacupuncture for the treatment of frozen shoulder: A systematic review and meta-analysis

**DOI:** 10.3389/fmed.2022.928823

**Published:** 2022-08-18

**Authors:** Jeong-Weon Heo, Jeong-Hun Jo, Jung-Ju Lee, Hee Kang, Tae-Young Choi, Myeong Soo Lee, Jong-In Kim

**Affiliations:** ^1^Department of Clinical Korean Medicine, Graduate School, Kyung Hee University, Seoul, South Korea; ^2^Humanitas College, Kyung Hee University, Yongin, South Korea; ^3^KM Science Research Division, Korea Institute of Oriental Medicine, Daejeon, South Korea

**Keywords:** frozen shoulder, electroacupuncture, systematic review, pain, acupuncture

## Abstract

**Background:**

Electroacupuncture (EA) has reportedly been successful in controlling pain, but there have been no systematic reviews examining the impact of EA on patients with frozen shoulder (FS). The purpose of this review is to provide evidence on the safety and efficacy of EA for pain management in patients with FS.

**Methods:**

We searched 11 databases from their inception: EMBASE, the Cochrane Library, PubMed, AMED, one Chinese medical database, and six Korean medical databases. Two researchers independently performed the study selection, data extraction, and assessment. Bias-related risk was evaluated using the Cochrane risk-of-bias assessment tool.

**Results:**

This review included thirteen studies involving 936 patients. The EA group exhibited improvements in FS pain (*MD* −1.11, 95% CI −1.61 to −0.61, *p* < 0.0001, *I*^2^ = 97%), function (SMD 2.02, 95% CI 0.36–3.69, *p* < 0.00001, *I*^2^ = 97%), and response rates (RR 1.16, 95% CI 1.07–1.25; *p* = 0.0002; *I*^2^ = 0%) over the manual acupuncture (MA) group. As an adjunct treatment, EA improved FS pain (SMD −1.12, 95% CI −1.52 to −0.71, *P* < 0.00001, *I*^2^ = 0) compared to the control treatments. No adverse effects were reported.

**Conclusion:**

EA is reported to improve FS pain and function compared with control treatments. Additionally, EA can be used as an adjunct therapy for FS pain. EA could emerge as a potent intervention against FS.

**Systematic review registration:**

[http://www.crd.york.ac.uk/PROSPERO/display_record.php?ID=CRD42021247090], identifier [CRD42021247090]

## Introduction

In the multifactorial disease frozen shoulder (FS), patients often have shoulder pain with limited active and passive mobility of the shoulder (1). The prevalence of frozen shoulder ranges from 2 to 5%, and most cases occur between the ages of 40 and 65 years (2, 3). The patient’s symptoms may appear suddenly and usually have a slow recovery (4). It takes anywhere between 1 and 4 years for FS to heal completely (5). The long morbidity period of the disease is burdensome for patients and profoundly affects their quality of life by causing issues such as sleep disturbance and restriction of daily activities (6).

For patients with FS, the use of intra-articular corticosteroids is linked with greater benefits than other interventions, including better pain reduction and range of motion (ROM) (7). However, the duration of this impact is limited (8). Acupuncture can serve as an alternative treatment. Acupuncture is mainly widely used in Asia for managing a variety of conditions, including cardiovascular diseases, infertility, pain and mental health (9–11). According to a meta-analysis of chronic pain (12), the effect of acupuncture did not decrease significantly over 12 months. Electroacupuncture (EA) is considered to enhance acupuncture-induced analgesia. It is possible that EA will have a lasting impact on FS with minimal side effects. In EA, a small current is passed through pairs of acupuncture needles. Needles are inserted into the same acupoints, and several pairs of needles are simultaneously stimulated. When standard operating procedures are followed, EA is a safe and easily sustained mode of treatment that does not exceed patients’ tolerance (13).

EA Analgesia, the mechanism by which EA controls pain, involves activation of the nervous system as well as induction of bioactive chemicals. Basically, EA treatment sends neuroimmune, and neuroinflammatory signals. In response to EA, sensory nerve fibers express calcitonin gene-related peptides and substance P, which bind to neurokinin 1 in mast cells, release serotonin, and exert analgesic effects (14). By activating the immune system, it also regulates the production of interleukin-2 (IL-2), interleukin-17 (IL-17), and interferon gamma (IFN-γ) through differentiation and activation of splenic T cells (15). In addition, treatment with EA inhibits sensory and affective components acting through peripheral, spinal, and supraspinal mechanisms. Bioactive molecules such as opioids, N/OFQ, serotonin, norepinephrine, glutamate receptors and transporters, cytokines, and signaling molecules play important roles. Opioids desensitize peripheral nociceptors, reduce the amount of proinflammatory cytokines in the periphery, and decrease cytokine and substance P levels in the spinal cord (16). In addition, the neurotransmitters serotonin and norepinephrine activate the descending inhibitory system, reduce GluN1 phosphorylation, and prevent pain (17). In addition, treatment with EA blocks the expression of inflammatory cytokines by releasing norepinephrine and acetylcholine from the adrenal gland *via* the HPA axis, sympathetic nervous system, and vagus nerve, creating a neuroimmune and neuroendocrine modulatory circuit. In addition, the HPA axis, sympathetic nervous system, and vagus nerve interact with immune cells and nociceptive neurons to create a feedback loop and suppress inflammation (18).

Although a meta-analysis on acupuncture for FS has been undertaken in the past (19), EA is not interchangeable with manual acupuncture (MA), since EA delivers stronger stimulation. One study (13) has shown that EA has greater analgesic effects than MA on several different types of pain. Therefore, pooling the results of MA and EA lowers the homogeneity of studies on acupuncture effects in systematic reviews (20). To the best of our knowledge, no systematic reviews on the impact of EA treatment for FS have been carried out. The purpose of this study was to review and meta-analyze the evidence from randomized controlled trials (RCTs) regarding the safety and efficacy of EA for pain management in patients with FS.

## Methods

This protocol was registered on PROSPERO (CRD42021247090) and published (5). The reporting of this review adheres to the recommendations of the Preferred Reporting Items for Systematic Reviews and Meta-Analyses (PRISMA) (21).

## Search strategy

The following electronic databases were searched from inception to June 2022: EMBASE, MEDLINE, CINII, the Cochrane Central Register of Controlled Trials (CENTRAL), one Chinese database [China National Knowledge Infrastructure (CNKI)], and six Korean databases [The Korean Traditional Knowledge Portal, KoreaMed, Oriental Medicine Advanced Searching Integrated System (OASIS), DBpia, the Research Information Service System, and the Korean Studies Information Service System]. We scanned the reference lists and retrieved any ongoing or recently completed studies that were not in the initial search results. Furthermore, the World Health Organization International Clinical Trials Registry Platform^1^ and Google Scholar^2^ were searched. To find unpublished trials, we searched the ClinicalTrials.gov registry.^3^ Our search strategy involved keywords such as “frozen shoulder,” “electroacupuncture,” “periarthritis of shoulder,” and “adhesive capsulitis” written in the languages of the databases (English, Chinese, Japanese, and Korean). Supplementary Material 1 lists the search terms for each database.

## Criteria for considering studies

### Types of studies

This review included only prospective RCTs. Observational studies, cohort studies, case series, case–control studies, uncontrolled trials, qualitative studies, and laboratory studies were excluded. No language restriction was imposed.

### Types of participants

Patients with FS were eligible regardless of age, sex, or race. We included only those studies that applied an external set of criteria to screen participants for FS.

### Types of interventions and controls

The review included studies assessing any form of invasive acupuncture with electrical stimulation. The control interventions could include many different treatments, such as general conventional care (drugs, exercise), MA, waiting-list conditions, or sham treatment (interventions that mimic “true” EA/true treatment but deviate in at least one element deemed important by EA theory, such as correct point location or skin penetration). The acceptability of sham acupuncture as a valid control is highly controversial (22–24), and we planned to analyze the results using subgroup and sensitivity analyzes. In this review, trials comparing EA plus another active treatment to the same active treatment in isolation were also included. However, RCTs wherein one type of EA was compared to another type were not included.

### Outcome measures

Pain intensity was the primary outcome measure. It was rated on a numerical rating scale and a visual analog scale (VAS). The secondary outcome measures were variables reflecting functional status [e.g., total effective rate, adverse effects (AEs), Constant–Murley score (CMS), and range of motion].

## Data collection, extraction, and assessment

### Study selection

Two independent reviewers (JWH and JHJ) searched and screened EMBASE, MEDLINE, CINII, CENTRAL, CNKI, and six Korean databases to find RCTs. Both the titles and the abstracts of the search results were screened, followed by an evaluation of criteria for study inclusion, after which the decisions were recorded in accordance with predefined criteria. The third reviewer (JIK, the corresponding author) resolved any lack of agreement or consensus in the study selection.

### Data extraction

Two reviewers (JWH and JHJ) read all the articles that remained after the above steps and extracted data from the articles based on predefined criteria. The data that were tabulated for future analysis included the following: name(s) of author(s), country where the study was performed, year of publication, age, sample size, sex distribution of participants, control intervention, EA intervention, main outcomes, and AEs. To create a summary table of findings, the Grading of Recommendations Assessment, Development, and Evaluation (GRADE) software was used to ascertain the quality of evidence on the basis of the Cochrane Handbook for Systematic Reviews of Interventions (25). When the reported data were unclear or insufficient, an author established contact with the first author or corresponding author of that paper by e-mail or telephone to seek clarity or request missing data.

### Evaluating risk of bias

The risk-of-bias assessment tool from the Cochrane Handbook for Systematic Reviews of Interventions was used to perform a quality assessment (26). The characteristics that were examined included generation of a random allocation sequence, concealment of allocation, blinding of both participants and personnel, blinding of the outcome assessment, incomplete data on outcomes, selective reporting of outcomes, and other sources of bias (baseline imbalance was evaluated). This review utilized “L,” “U,” and “H” as grades for these assessments, where “U” (“unclear”) indicated that the risk of bias was uncertain, “H” (“high”) indicated a high risk of bias, and “L” (“low”) indicated a low risk of bias. In the event of disagreement, the authors reached a consensus by discussion. Information about the risk-of-bias assessment for the aforementioned studies is presented in a table. A critical discussion of the results and their implications is provided.

## Data analysis

Cochrane Collaboration’s software Review Manager (RevMan), v. 5.4.1 for Windows (The Nordic Cochrane Center, Copenhagen, Denmark) was used to conduct all statistical analyses. This was followed by an evaluation of the differences between the intervention and control groups. In the analysis of clinical efficacy, the assessment of categorical data considered the risk ratios and continuous data with respect to the mean difference (MD). Both categorical and continuous variables are expressed as efficacy values with 95% confidence intervals (CIs). In cases where outcome variables had different scales, the standardized MD was preferred over the weighted MD. If heterogeneity (defined by the results of statistical heterogeneity testing, with *p* < 0.1 on the chi-square test and Higgins’ *I*^2^ ≥ 50%) was detected, the cause of clinical heterogeneity was assessed by performing subgroup analyses. To assess combined effect sizes from efficacy variables, we used a random-effects model. Notably, we expected substantial clinical heterogeneity across the studies that were included based on the study designs, the diversity of interventions, and other conditions. If more than ten studies were available, publication bias was evaluated by drawing funnel plots (27).

## Results

### Search results

**FIGURE 1:**
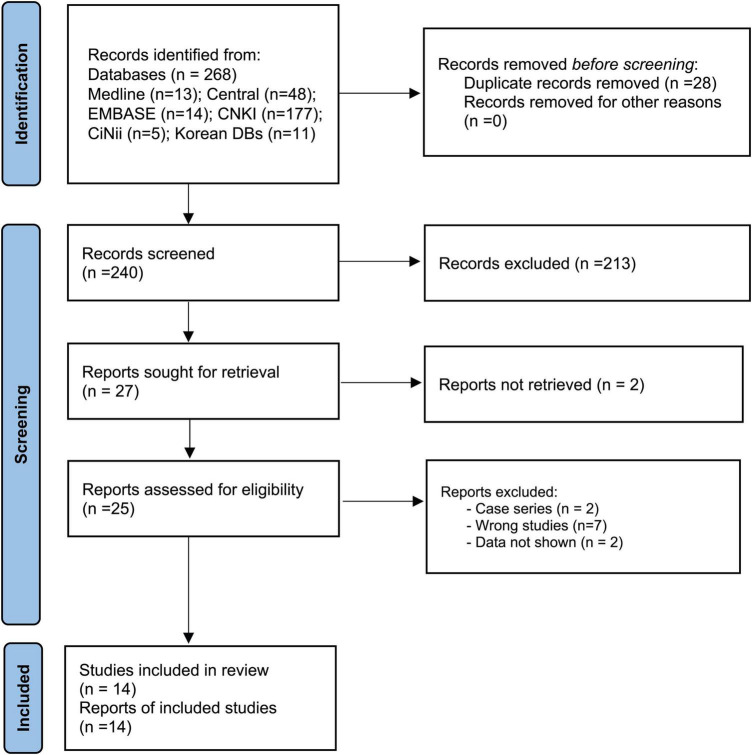
Study flow chart. A flowchart of the patient selection process.

The 11 database searches yielded 268 studies. Fourteen studies (28–41) met the inclusion criteria (Figure 1). Tables 1, 2 shows the primary features of the 14 included studies.

**TABLE 1:** Summary of the characteristics of the included studies.

References	FS duration Sex (M/F) Age	Intervention	Control	Outcome measures	Main results	Authors’ conclusion
Lin et al. (28)	A: 52.1; B: 52.9 days A: 16/24; B: 18/22 A: 55.4; B: 57.3	(A) EA (30 min, *n* = 40)	(B) MA (30 min, *n* = 40)	1) Pain (VAS^†^) 2) Function (Constant–Murley^‡^) 3) Response rate	1) MD −0.65 [−1.25, −0.05], *P* < 0.05 2) MD 7.45 [4.58, 10.32], *P* < 0.001 3) RR 1.19 [1.00, 1.41], *P* < 0.05	“EA…reduces shoulder pain, improves shoulder joint mobility, …”
Shao (29)	A: 6.85; B: 6.82 days A: 13/16; B: 12/17 A: 54.5; B: 53.8	(A) EA (30 min, *n* = 20)	(B) MA (30 min, *n* = 29)	Response rate	RR 1.12 [0.95, 1.32], NS	“EA has a reliable curative effect…”
Shi et al. (30)	A: 3.11; B: 2.97 months A: 23/34; B: 18/38 A: 54.9; B: 52.1	(A) EA (30 min, *n* = 57)	(B) MA (30 min, *n* = 56)	1) Pain (VAS^†^) 2) Function (neck and shoulder pain^‡^) 3) Response rate	1) MD −1.50 [−1.57, −1.43], *P* < 0.001 2) MD 17.87 [16.49, 19.25], *P* < 0.001 3) RR 1.18 [1.01, 1.38], *P* < 0.05	“…EA… [is] superior…”
Cong et al. (31)	A: 2.66; B: 2.49 months A: 14/13; B: 10/18 A: 56; B: 52	(A) EA (30 min, *n* = 27)	(B) MA (30 min, *n* = 28)	1) Pain (VAS^†^) 2) Function (neck and shoulder pain^‡^) 3) Response rate	1) MD −1.61 [−1.77, −1.45], *P* < 0.001 2) MD 15.48 [11.32, 19.64], *P* < 0.001 3) RR 1.17 [0.97, 1.41], NS	“…EA…[was] better than MA”
Cong et al. (32)	A: 2.66; B: 2.05 months A: 11/11; B: 17/11 A: 52.3; B: 55.0	(A) EA (60 min, *n* = 22)	(B) MA (60 min, *n* = 28)	1) Pain (VAS^†^) 2) Function (neck and shoulder pain^‡^) Response rate	1) MD −0.56 [−0.73, −0.39], *P* < 0.001 2) MD 4.05 [−2.29, 10.39], NS 3) RR 1.11 [0.89, 1.38], NS	No clear conclusion given
Huang (33)	n.r. A: 13/14; B: 10/11 A: 56.2; B: 54.5	(A) EA, plus B (*n* = 27)	(B) Joint mobilization (*n* = 21)	1) Pain (VAS^†^) 2) Response rate	1) MD −1.21 [−1.94, −0.48], *P* < 0.01 2) RR 1.06 [0.91, 1.25], NS	“…[the relationship of EA]… with joint mobilization…is definite.”
Yang et al. (34)	1–6 months n.r. n.r.	(A) EA, plus B (*n* = 31)	(B) Arthrolysis (*n* = 31)	1) Pain (MRMC^‡^) 2) Function (MRMC^‡^) 3) Response rate	1) MD 7.82 [4.78, 10.86], *P* < 0.001 2) MD 5.79 [4.72, 6.86], *P* < 0.001 3) RR 1.03 [0.92, 1.16], NS	“…arthrolysis… with EA … is better…”
Li et al. (35)	n.r. n.r. n.r.	(A) EA, plus B (*n* = 31)	(B) general rehabilitation (*n* = 31)	1) Pain (VAS^†^) 2) Function (aROM^‡^) 3) Response rate	1)-2) details n.r., *P* < 0.05 3) RR 1.36 [1.08, 1.72], *P* < 0.01	“…[general rehabilitation] combined with EA is beneficial.”
Huang et al. (36)	A: 6.1; B: 6 months A: 32/20; B: 14/12 n.r.	(A) EA, plus B (*n* = 52)	(B) TDP (*n* = 26)	Response rate	RR 1.05 [0.95, 1.15], NS	“…EA… with TDP…is better than…TDP…alone”
He (37)	A: 4.03; B: 3.85 months A: 18/14; B: 14/16 A: 48.1; B: 49.0	(A) EA, plus B (*n* = 32)	(B) Intermediate frequency (4∼6 kHz) (*n* = 30)	Response rate	RR 1.32 [1.06, 1.65], NS	“EA…with intermediate frequency has a significant curative effect”
Ke et al. (38)	A: 3.3; B: 3.6 weeks A: 8/22; B: 9/21 A: 48.4; B: 50.2	(A) EA, plus B (*n* = 30)	(B) Very high frequency + joint mobilization (*n* = 30)	1) Function (Constant–Murley^‡^) 2) Response rate	1) MD 17.30 [6.59, 28.01], *P* < 0.01 2) RR 1.33 [1.04, 1.72], *P* < 0.05	“Treatment…with EA …improved [outcomes].”
Li et al. (39)	n.r. A: 14/16; B: 15/15 n.r.	(A) EA, plus B (*n* = 30)	(B) Electromoxibustion (Fuyang pot warming) (*n* = 30)	1) Pain (VAS^†^) 2) Function (Constant–Murley^‡^; ASES^‡^) 3) Response rate	1)-2) details n.r., *P* < 0.01 3) A vs. B: RR 1.08 [0.88, 1.32], NS	“Fuyang pot warming…with EA…has…[a greater] curative effect … than EA… or Fuyang pot warming therapy alone”
Huang et al. (41)	A: 6.53; B: 7.03 months A: 13/17; B: 11/19 A: 52.0; B: 53.6	(A) EA, plus B (*n* = 30)	(B) Tuina (*n* = 30)	1) Function (Constant–Murley^‡^) 2) Response rate	1) MD 11.80 [8.72, 14.88], *P* < 0.001 2) RR 1.50 [1.09, 2.06], *P* < 0.01	“EA… with tuina… [was] superior… [for] improving range of motion… [and] alleviating clinical symptoms”
Li (40)	A: 7.85; B: 7.85 days A: 29/45; B: 32/42 A: 54.4; B: 50.2	(A) EA, plus B (*n* = 74)	(B) Tuina (*n* = 74)	Response rate	RR 1.13 [1.01, 1.27], *P* < 0.05	“Tuina… [with] EA has a reliable curative effect”

^†^A lower score indicates better condition; ^‡^a higher score indicates better condition.

A, intervention group; B, comparison group.

ASES, American Shoulder and Elbow Surgeons; AEs, adverse effects; EA, electroacupuncture; FS, frozen shoulder; MA, manual acupuncture; MD, mean difference; MRMC, Michael Reese Medical Center; n.r., not reported; NS, not significant; RCT, randomized controlled trial; aROM, active range of motion; RR, risk ratio; SD, standard deviation; TDP, tending diancibo pu; VAS, visual analog scale.

**TABLE 2:** Summary of the regimens used in the included studies.

References	Acupuncture points	Medium (model, manufacturer)	Wave (Hz)	Intensity	Treatment session and interval
Lin et al. (28)	EA: Ashi points, LI15, TE14, EX-UE70, SI9, LI11, TE5, LI4, SI3 MA: Ashi points, LI15, TE14, EX-UE70, SI9, LI11, TE5, LI4, SI3	n.r.	Dense-dispersed wave (2 Hz/100 Hz)	Tolerance level	14 times (once daily)
Shao (29)	EA: EX-UE70, LI15, TE14, SI11, LI4, GB34 MA: X-UE70, LI15, TE14, SI11, LI4, GB34	n.r.	n.r.	n.r.	20 times (once daily)
Shi et al. (30)	EA: EX-UE70, TE14, LI15, SI10, TE5, LI MA: EX-UE70, TE14, LI15, SI10, TE5, LI4	HANS LH-202H	Dense-dispersed wave (2 Hz/100 Hz)	(3 ± 2) mA	5 times (once every other day)
Cong et al. (31)	EA: EX-UE70, LI15 or TE14, SI10, TE5, LI4 MA: EX-UE70, LI15 or TE14, SI10, TE5, LI4	HANS LH-202H	Dense-dispersed wave (2 Hz/100 Hz)	n.r.	5 times (once every other day)
Cong et al. (32)	EA: EX-UE70, LI15, TE14, SI10, TE5, LI4 MA: EX-UE70, LI15, TE14, SI10, TE5, LI4	HANS LH-202H	Dense-dispersed wave (2 Hz/100 Hz)	(1 ± 2) mA	5 times (once every other day)
Huang (33)	(Ashi points, LI15, TE14, LI11, LI4/Flexion restriction: LI14, Tai Jian Extension restriction: TE13, SI10 Abduction restriction: Nao Shang, Cheng Feng External rotation restriction: SI11, SI9 Internal rotation restriction: Jian Nei Ling) + joint mobilization	n.r.	Continuous (high frequency 10 min − > low frequency 10 min)	n.r.	10 times (once daily)
Yang et al. (34)	(LI15, SI9, EX-UE70, LI14, PC3, LI11, LU5, GB34, SP8, SI11, TE5/GB12, BL60, LI4, ST38, TE3, LU5, LU7, TE5)	n.r.	Dense-dispersed wave (2 Hz/15 Hz OR 100 Hz)	n.r.	5 times (once daily)
Li et al. (35)	(LI meridian, SI meridian, TE meridian, LI15, Ashi points, TE14)	KWD808-I	Continuous (0–10 Hz)	2–4 mA	20 times (once daily)
Huang et al. (36)	(Ashi points, LI15, SI9, TE14, EX-UE70)	G6805	2–20 Hz	2–3 mA	10 times (once daily)
He (37)	(Ashi points, LI15, EX-UE70, SI9, LI11, LI4, SP9, LI3, TE3, SI3)	G6805	n.r.	Tolerance level	20 times (once daily)
Ke et al. (38)	SI14, EX-UE70, LI15, TE14, SI9, LI11, TE5, LI4 + Very high frequency (frequency 40.68 mHz, wavelength 7.37 m, output 200 W)	G6805-II	Dense-dispersed wave (50 Hz/100 Hz)	n.r.	21 times (once daily)
Li et al. (39)	(Ashi points, GB20, GV14, GB21, LI15, LI14, ST39, BL57) + Fuyang pot warming (Ashi points, GB20, GV14, GB21, LI15, SI9, LU1, SI11)	HANS LH-202H	Dense-dispersed wave (2 Hz/100 Hz)	1–1.5 mA	12 times (three times/week)
Huang et al. (41)	EA: Ashi points, LI15, TE14, EX-UE70, SI9	SDZ II, Hwato	Dense-dispersed wave (n.r.)	Tolerance level	21 times (once daily)
Li (40)	(SI9, Tai jian, LI15, TE14, SI11, LI4)	n.r.	n.r.	n.r.	20 times (once daily)

EA, electroacupuncture; MA, manual acupuncture; n.r., not reported.

### Included studies’ characteristics

All of the included studies (28–41) were carried out in China. Thirteen studies were written in Chinese (28, 29, 31–41), and only one study (30) was published in English.

Five studies (28–32) compared EA with MA. Three studies compared EA plus Western medicine (WM) to WM in isolation, including joint mobilization (33), arthrolysis (34) and general rehabilitation (35). Two studies compared EA plus frequency therapy (FT) to FT in isolation, including TDP (36) and intermediate frequency therapy (37). One study (38) compared EA plus ultra-short-wave therapy with joint mobilization to ultra-short-wave therapy with joint mobilization. Three studies compared EA plus CM with CM in isolation, including electromoxibustion (EM) (39) and tuina (40, 41). Eight studies (28, 30–32, 34, 38, 39, 41) utilized dense-dispersed waves for EA, five studies (28, 30–32, 39) applied 2 Hz/100 Hz, one study (38) applied 50 Hz/100 Hz, one study (34) applied 2 Hz/15 Hz or 100 Hz, two studies (33, 35) used a continuous frequency, and one study (36) applied 2–20 Hz (Table 2). No mention of the frequency was made in the other four articles (29, 37, 40, 41).

### Risk of bias

**FIGURE 2:**
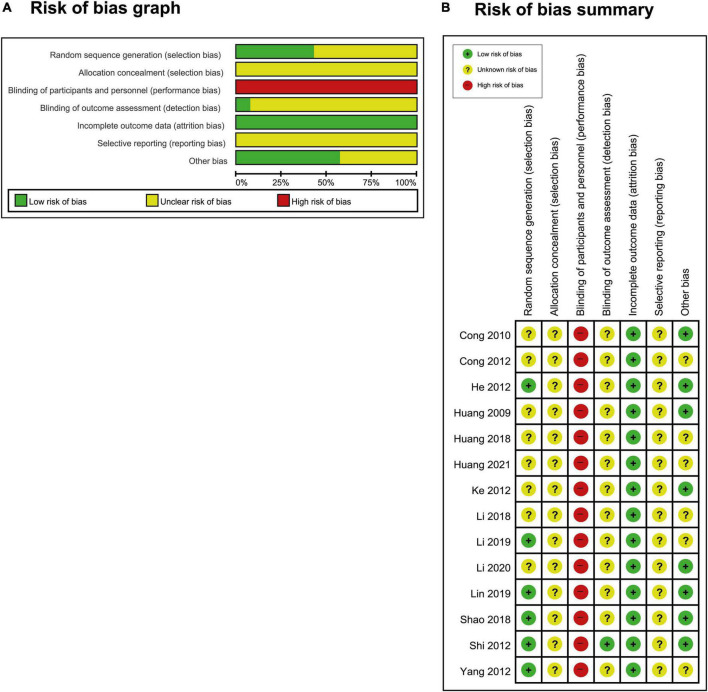
**(A)** Risk-of-bias graph and **(B)** risk-of-bias summary: The present authors’ judgments regarding the risk of each form of bias in all included studies.

Six studies (28, 29, 32, 33, 35, 40) utilized a random number table, and their risk of bias from random number sequence generation was found to be low (Figure 2). Eight studies (31–33, 35, 36, 38, 40, 41) did not clarify whether they employed a random number generation method, which means that they had an ambiguous risk of bias for the generation of random allocation. All studies (28–41) failed to describe the method of allocation concealment and thus had an unclear risk of bias in this respect. The investigator did not perform blinding in one study (30); however, it was determined that blinding would not impact the assessment of results, and so the risk of detection bias was low. As for the other 13 studies (28, 29, 31–41), a decision could not be made as to whether the outcome assessor was blinded. For this reason, these studies had an ambiguous risk of detection bias. Twelve studies (28, 29, 31–38, 40, 41) were found to have a low risk of attrition bias owing to low dropout rates. Higher dropout rates were reported in two papers (30, 39), but they did not impact the results; for this reason, the risk of attrition bias was considered low. Notably, due to the lack of registered protocols, all studies (28–41) were found to have an unclear risk of reporting bias; these studies may not have prespecified the variables of interest and the anticipated values of these variables. Eight studies (28–31, 36–38, 40) were found to have a low risk of other forms of bias due to the absence of a significant difference in baseline information between the groups. The other six studies (32–35, 39, 41) had an ambiguous risk of other forms of bias due to the potential for additional biases; the articles did not provide sufficient information to rule out this possibility.

### Effect of interventions

#### Electroacupuncture vs. manual acupuncture

**FIGURE 3:**
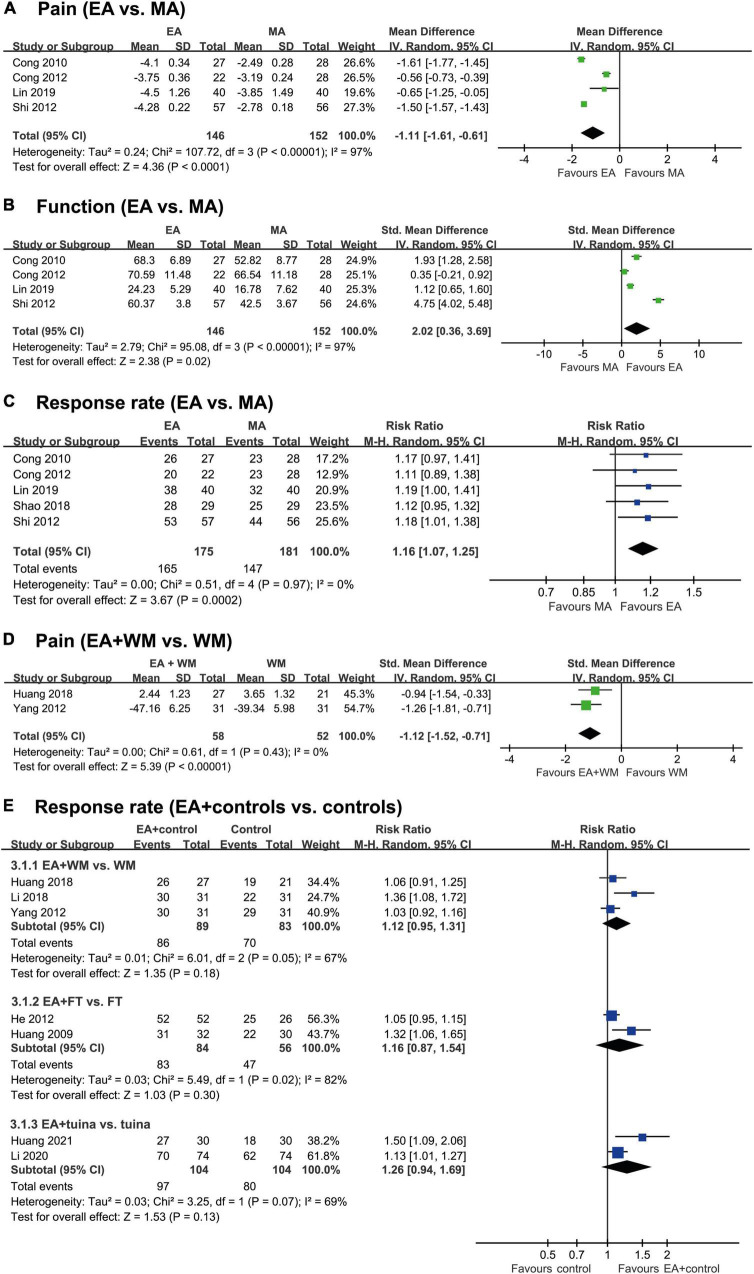
Forest plot of each outcome according to the comparison made. **(A)** Pain (EA vs. MA), **(B)** Function (EA vs. MA), **(C)** Response rate (EA vs. MA), **(D)** Pain (EA + WM vs. WM) and **(E)** Response rate (EA + controls vs. controls). EA, electroacupuncture; MA, manual acupuncture; FT, frequency therapy; WM, Western medicine.

Five RCTs compared the effects of EA and MA on FS symptoms (28–32). Four RCTs (28, 30–32) compared the effects of EA and MA on VAS pain scores, and the meta-analysis showed that EA effectively reduced pain (*MD* −1.11, 95% CI −1.61 to −0.61, *p* < 0.0001, Figure 3A), although there was high heterogeneity (*I*^2^ = 97%).

Three RCTs (28, 30, 31) also reported the positive effects of EA over MA on the improvement of function, whereas one RCT (32) failed to do so. Our meta-analysis showed favorable effects of EA on the improvement of FS function (SMD 2.02, 95% CI 0.36–3.69, *p* = 0.02, Figure 3B), but there was a high degree of heterogeneity (*I*^2^ = 97%).

Four RCTs (28, 29, 31, 32) showed equivalent effects of EA and MA on the response rate, and one RCT (30) showed positive effects of EA compared to MA. Meta-analysis showed favorable effects of EA on the response rate (RR 1.16, 95% CI 1.07–1.25, *p* = 0.0002, *I*^2^ = 0%; Figure 3C).

#### Electroacupuncture plus western medicine vs. western medicine

Three RCTs tested the effects of EA plus WM compared with WM alone on the symptoms of FS. Two of them (33, 34) reported positive effects of EA plus WM on pain, and the meta-analysis also showed favorable effects of EA plus WM (SMD −1.12, 95% CI −1.52 to −0.71, *p* < 0.00001, *I*^2^ = 0%; Figure 3D). Only one RCT assessed the effects of EA plus MA on function; that study reported positive effects of EA plus MA.

One RCT (35) showed a favorable effects of EA plus WM on response rate, while the other 2 RCTs (33, 34) failed to do so. The meta-analysis also failed to show any superior effect of EA plus WM on the response rate (RR 1.12 95% CI 0.95–1.31, *p* = 0.18, *I*^2^ = 67%; Figure 3E).

#### Electroacupuncture plus frequency therapy vs. frequency therapy

Two RCTs (36, 37) tested the effect of EA plus FT compared with FT alone on the response rate. The meta-analysis failed to show a superior effect of EA plus FT on the response rate (RR 1.16, 95% CI 0.87–1.54, *p* = 0.30, Figure 3E). These studies had high heterogeneity (*I*^2^ = 82%).

One RCT (38) investigated the effect of EA plus FT and joint mobilization compared to FT plus joint mobilization alone on function and response rate. The results showed favorable effects of EA plus FT and JM on both function and response rate.

### Electroacupuncture plus CM therapies vs. CM therapies

Three RCTs compared FS symptoms after EA plus CM vs. CM alone. The CMs included EM (39) and tuina (40, 41). EA plus EM showed positive effects on function and pain but not on response rate compared with EM alone. EA plus tuina showed positive effects of EA on function and response rate compared with tuina alone. However, the meta-analysis failed to show a superior effect of EA plus tuina on the response rate (RR 1.26, 95% CI 0.94–169, *p* = 0.13, Figure 3E). These studies had high heterogeneity (*I*^2^ = 69%).

### Safety of interventions

Thirteen studies failed to report whether any AEs took place (28–34, 36–41). An absence of AEs was reported by Li et al. (35). Since 13 of the 14 studies failed to record whether any AEs took place, more studies are needed regarding the safety of EA.

### Summary of findings

The certainty of the evidence comparing the impacts of EA and MA on pain was downgraded from high to very low (three levels) owing to the severity of concerns about imprecision, inconsistency, and risk of bias (Table 3). The certainty of the evidence comparing the impacts of EA and MA on function was downgraded from high to very low (three levels) owing to the severity of concerns regarding imprecision, inconsistency, and risk of bias. The certainty of the evidence comparing the impacts of EA and MA on response rates was downgraded from high to low (two levels) owing to the severity of concerns pertaining to imprecision and the risk of bias. The certainty of the evidence that compared the impact of EA treatment plus WM vs. WM alone on pain was downgraded from high to low (two levels) owing to serious concerns pertaining to the risk of bias and imprecision.

**TABLE 3:** Summary of findings.

Patient or population: Patients with FS Intervention: EA or EA + WM Comparison: MA or WM					
Outcomes	Anticipated absolute effects* (95% CI)		Relative effect (95% CI)	No of participants (Studies)	Certainty of the evidence (GRADE)
	Risk with MA	Risk with EA			
**EA vs. MA**					
Pain (VAS)	The mean pain (as measured by the VAS) ranged from −1.61 to −0.61	MD 1.11 lower (1.61 lower to 0.61 lower)	–	298 (4 RCTs)	⊕⁣○⁣○⁣○ Very low^a,b,c^
Function	–	SMD 2.02 SD higher (0.36 higher to 3.69 higher)	–	298 (4 RCTs)	⊕⁣○⁣○⁣○ Very low^a,b,c^
Response rate	812 per 1,000	942 per 1,000 (869–1,000)	RR 1.16 (1.07–1.25)	356 (5 RCTs)	⊕⊕⁣○⁣○ Low^a,d^
**EA + WM vs. WM**					
Pain intensity	–	SMD 1.12 SD lower (1.52 lower to 0.71 lower)	–	110 (2 RCTs)	⊕⊕⁣○⁣○ Low^a,b^

^a^Most information is unclear (random number generation and allocation concealment).

^b^Serious limitation of inconsistency: Unexplained high heterogeneity (I^2^ > 50%).

^c^Total participants < 400.

^d^Total participants < 300.

*The risk in the intervention group (along with its 95% confidence interval) is based on the assumed risk in the comparison group and the relative effect of the intervention (and its 95% CI). CI, confidence interval; MD, mean difference; RR, risk ratio; SMD, standardized mean difference; WM, Western medicine.

GRADE Working Group grades of evidence. High certainty: we are very confident that the true effect lies close to the estimate of the effect. Moderate certainty: we are moderately confident in the effect estimate: the true effect is likely to be close to the estimate of the effect, but there is a possibility that it is substantially different. Low certainty: our confidence in the effect estimate is limited: the true effect may be substantially different from the estimate of the effect. Very low certainty: we have very little confidence in the effect estimate: the true effect is likely to be substantially different from the estimate of effect.

## Discussion

EA was found to be an efficacious method for treating FS in this review. The meta-analysis showed that EA led to a greater reduction of FS pain than MA did, although with a very low certainty of evidence. In comparison to MA, EA led to a superior degree of functional improvement in FS patients, with a very low certainty of evidence. In comparison to MA, EA enhanced the response rate of FS, again with a low certainty of evidence. Compared with WM used in isolation, EA plus WM reduced FS pain with a low certainty of evidence. Four RCTs compared the efficacy of EA and MA as treatments for FS (29, 33, 36, 39). Three RCTs reported favorable impacts on functional scores in FS patients treated with EA compared to those treated with MA (29, 30, 36). However, one trial showed no favorable effects of EA on function compared to MA (32), which might be attributable to the treatment duration of 60 min. Generally, the longer the stimulation time, the more effective the stimulation. A shorter stimulation time translates to less effective stimulation. However, if the stimulation time is extended indefinitely, the effective stimulation worsens or becomes invalid (42). Therefore, EA may have led to functional improvement in FS patients if an appropriate treatment time had been set. Additionally, when using EA for FS, it would be prudent to enhance the patient’s function by selecting a target treatment area that would focus on the muscles impacting the shoulder joint’s ROM, for example, the elevator muscle of the scapula, as opposed to considering only the Ashi points. Therefore, in future RCTs exploring the use of EA for FS, it could be worthwhile to choose the treatment area by focusing on the muscle that is restricting shoulder movement.

This review included only one study where AEs were linked with EA. Despite being a relatively safe treatment tool, EA is not free of risks. Therefore, it is necessary to conduct more studies to evaluate the risk of AEs.

According to our assessment, allocation concealment, appropriate randomization, blinding of outcome assessment, and selective reporting were not mentioned in the various RCTs included in this review. Notably, random number generation was reported in only six studies (28, 29, 32, 33, 35, 40), and the blinding of the outcome assessment was referred to in only one study (30). This indicates a high risk of bias in all the included studies, which could potentially result in false positives. Moreover, since all the studies (28–41) were performed in China, it is important to conduct independent studies in different countries to determine the generalizability of the results.

In terms of the therapeutic effect for FS, no systematic review thus far has focused only on EA. One systematic review (19) examined the impacts of acupuncture on FS in five studies (30, 43–46). EA was utilized as an intervention in some of those studies, but three (44–46) were excluded from the present study because they failed to satisfy its inclusion criteria, and no meta-analysis was carried out on the other two studies (30, 43). Thus, no strong recommendation can be made for the use of EA in FS. Unlike prior reviews, ours has demonstrated the efficacy of EA in FS compared with MA. Furthermore, the efficacy of EA as an adjunct therapy for FS has been established.

This study has certain limitations. First, since the sample size of the meta-analysis was small, one must be cautious about generalizing the results. The reason for the small sample size was because the trials used inconsistent outcome measures, with only a small number of trials actually being eligible for the meta-analysis of each measure. Second, the treatment regimen used in different trials varied in several aspects, such as the selection of acupuncture points and the treatment frequency. Thus, future studies to assess treatment effects should use a consistent acupuncture treatment regimen. Third, our study’s findings should be interpreted cautiously owing to the high risk of bias within the included studies. The fourth limitation is the high heterogeneity between the included trials. The included studies used different standards to measure effectiveness through the response rate, and the clinical characteristics of the patients and their treatments were also different, including the dose and type of intervention and origin of shoulder pain. The pooled results also showed high statistical heterogeneity. Clearly, these potential confounding factors may reduce the comparability of the final results; therefore, the results need to be interpreted with caution.

To enable RCTs and pilot trials designed as precursors for appropriate RCTs, future studies on FS treatment with EA should emphasize appropriate and uniform methods. It will also be necessary to conduct long-term studies to determine the duration of the treatment effects. Furthermore, a cost analysis needs to be conducted.

In conclusion, the results from this systematic review and meta-analysis suggest that EA is more effective than MA for managing FS, with larger effect sizes in terms of pain (particularly after 30 min of treatment), function, and response rate. Additionally, this systematic review and meta-analysis provides suggestive evidence for the superiority of EA as an adjunct therapy to reduce FS pain. However, given the high risk of bias, the differences in treatment regimens, and the small sample size, the level of evidence is low. To confirm the effect of EA on FS, it will be necessary to conduct well-designed research studies with larger sample sizes.

## Data availability statement

The original contributions presented in the study are included in the article/Supplementary material, further inquiries can be directed to the corresponding author/s.

## Author contributions

J-IK: conceptualization. J-WH and J-HJ: data curation and methodology. J-WH, J-HJ, J-JL, and J-IK: formal analysis. J-WH, J-HJ, HK, and T-YC: investigation. MSL and J-IK: project administration. J-JL and HK: resources. J-WH, MSL, and J-IK: software. HK, T-YC, MSL, and J-IK: supervision. J-WH: writing—original draft. J-WH, J-HJ, J-JL, HK, T-YC, MSL, and J-IK: writing—review and editing. All authors read and approved the final manuscript.

## Footnotes

1
http://apps.who.int/trialsearch/

2
http://scholar.google.co.kr/

3
https://clinicaltrials.gov/ct2/home
