# Supplementary material for: Electroacupuncture for the treatment of frozen shoulder: A systematic review and meta-analysis
Source: Front Med (Lausanne). 2022 Aug 18;9:928823. doi: 10.3389/fmed.2022.928823 (PMC9433806; doi:10.3389/fmed.2022.928823)
Supplement: Supplementary file 1 [file Data_Sheet_1.PDF]

Supplementary 1. Search strategies

A. MEDLINE

1. Frozen Shoulder/
2. Adhesive Capsulitis/
3. Shoulder Periarthritis/
4. (((frozen[TIAB] OR adhesive[TIAB] OR Capsulitis[TIAB] OR Bursitis[TIAB] OR Bursitides[TIAB] OR periarthritis[TIAB] OR peri-arthritis[TIAB] OR periarthritides[TIAB] OR pericapsulitis[TIAB] OR stiff*[TIAB]))) AND (shoulder*[TIAB])
5. #1 OR #2 OR #3 OR #4
6. electroacupuncture[TIAB] OR ‘electric stimulation’[TIAB] OR ‘electrical stimulation’[TIAB]
7. #5 and #6
8. randomized controlled trial [PT]
9. controlled clinical trial [PT]
10. randomized [TIAB]
11. placebo [TIAB]
12. clinical trials as topic [mesh: noexp]
13. randomly [TIAB]
14. trial [TI]
15. #8 OR #9 OR #10 OR #11 OR #12 OR #13 OR #14
16. animals [mh] NOT humans [mh]
17. #15 NOT #16
18. #7 AND #17

B. CENTRAL

1. MeSH descriptor: [Bursitis] explode all trees
2. Frozen Shoulder: ti,ab,kw
3. Adhesive Capsulitis: ti,ab,kw
4. Shoulder Periarthritis: ti,ab,kw
5. (frozen or adhesive or capsulitis or bursitis or bursitides or periarthritis or peri-arthritis or periarthritides or pericapsulitis or stiff) near/5 (Shoulder*):ti,ab,kw (Word variations have been searched)
6. #1 OR #2 OR #3 OR #4 OR #5
7. electroacupuncture OR ‘electric stimulation’ OR ‘electrical stimulation’:ti,ab,kw (Word variations have been searched)
8. #6 and #7

C. EMBASE

1. ’Frozen Shoulder’/exp
2. ’Adhesive Capsulitis’/exp
3. ’Shoulder Periarthritis’/exp
4. ((frozen OR adhesive OR Capsulitis OR Bursitis OR Bursitides OR periarthritis OR peri-arthritis OR periarthritides OR pericapsulitis OR stiff*) NEAR/5 (Shoulder*)):ab,ti
5. #1 OR #2 OR #3 OR #4
6. (electroacupuncture OR ‘electric stimulation’ OR ‘electrical stimluation'):de,ab,ti
7. #5 and #6
8. 'crossover procedure':de OR 'double-blind procedure':de OR 'randomized controlled trial':de OR 'single-blind procedure':de OR (random* OR factorial* OR crossover* OR cross NEXT/1 over* OR placebo* OR doubl* NEAR/1 blind* OR singl* NEAR/1 blind* OR assign* OR allocat* OR volunteer*):de,ab,ti
9. #7 and #8

D. CNKI

1. SU=('肩周炎'+'粘連性關節囊炎'+' 五十肩') AND SU=(随机) AND SU=('電針’)
2. AB=('肩周炎'+'粘連性關節囊炎'+' 五十肩') AND AB=(随机) AND AB=('電針’)
3. TI=('肩周炎'+'粘連性關節囊炎'+' 五十肩') AND TI=(随机) AND TI=('電針’)

E. CiNii

1. (五十肩 OR ‘frozen shoulder*’ OR ‘adhesive capsulitis’’ OR ‘shoulder periarthritis’) AND (電気鍼 OR ‘electroacupuncture’ OR ‘electric stimulation’ OR ‘electrical stimulation’)

F. Korean DB

1. (동결견 OR 유착성관절낭염 OR 오십견 OR ‘frozen shoulder*’ OR ‘adhesive capsulitis’ OR ‘shoulder periarthritis’) AND (전침 OR 전기침 OR electroacupuncture OR ‘electric stimulation’ OR ‘electrical stimulation’)
